# Supplementary material for: Are carbon emissions trading and green financial instruments synergistic? -Comprehensive quantitative research based on content analysis
Source: PLoS One. 2024 Mar 7;19(3):e0298601. doi: 10.1371/journal.pone.0298601 (PMC10919601; doi:10.1371/journal.pone.0298601)
Supplement: S1 Data — (ZIP) [file pone.0298601.s001.zip › Data/地级市Z_F工作报告.zip!/╕╩╦α/┬ñ─╧╩╨/└┤╫╘╣1⁄2╓┌║┼í╛┬φ┐╦╩2╛▌═°í┐_2013.docx]

各位代表：

现在，我代表市人民政府向大会作工作报告，请予审议，并请各位政协委员和列席人员提出意见。

2012年工作回顾

2012年，面对宏观经济下行压力加大的严峻形势，我们在市委的坚强领导下，在市人大、市政协的监督支持下，认真贯彻落实省第十二次党代会和市第三次党代会精神，求真务实，艰苦创业，圆满完成了全年各项目标任务，经济社会保持平稳较快发展。全市完成生产总值226亿元，比上年增长13.1%；完成固定资产投资410亿元，增长41.1%；完成大口径财政收入33.9亿元、公共财政预算收入16亿元，分别增长24.6%和22.6%；完成社会消费品零售总额57.4亿元，增长17.5%；城镇居民人均可支配收入14077元，增长16.1%；农民人均纯收入3088元，增长17.8%。

一、政策和项目争取取得新成果，固定资产投资保持大幅增长。进一步加大政策和项目争取力度, 市委、市政府主要领导多次带领有关部门上省赴京衔接争取项目。在省委、省政府和省直厅局的关心支持下，经过市委、市政府积极争取，省政府出台了《关于支持陇南市加快经济社会发展的意见》，省直有关厅局分别出台了行业支持意见。秦巴扶贫实施规划和生态文明示范市得到批复，“两江一水”综合治理规划即将获得批复。国家发改委、财政部追加下达我市灾后重建项目征地补助资金12.5亿元。武九高速公路纳入国家高速公路网规划，成州民用机场开工建设。全力支持重大项目建设，征地拆迁、水电路等问题及时得到协调解决，在建各类重大项目进展顺利，兰渝铁路、武罐高速、成武高速公路当年完成投资87.2亿元。市列重点项目累计完成投资210亿元，武都拱坝河电站、徽县泥阳现代农业综合示范园、三森太阳能光伏发电等重点项目进展良好。成县“8.12”灾后重建任务全面完成。招商引资迈上新台阶,完善了招商引资工作机制，各级领导和部门负责人带头招商，签约了一批亿元以上招商引资项目，全年签订合同项目135个，签约资金237亿元，到位资金91.3亿元，同比增长65%。在灾后重建任务完成后，全市固定资产投资仍然保持了强劲的增长势头。

二、工业经济快速发展，规模效益不断提升。针对铅锌等主导工业产品价格持续低迷、硅铁企业生产困难的实际，依托资源优势，引进战略投资，加大黄金等资源开发力度，加强工业经济运行监测服务，工业经济在困境中逆势上扬。全市规模以上工业完成增加值35.3亿元，同比增长21.5%，增幅居全省第四。实施市列重点工业项目30项，累计完成投资29.7亿元。西和大桥金矿、后川坝金矿、两当金润玉石材等13个项目顺利建成，紫金矿业黄金采选、厂坝公司矿山扩能改造、金徽矿业郭家沟铅锌采选、金徽酒业优质酒酿造扩能等一批亿元以上重点项目开工建设，工业经济发展后劲不断增强。加快培育成长性好、发展潜力大的中小企业，两当招金矿业、成县华龙恒业公司、文县祁连水泥等15户企业进入规模以上企业行列。有色金属采选、冶炼，水泥、医药、农副产品加工等重点行业实现平稳较快发展。徽县工业集中区整体纳入西成经济开发区扩区规划编制完成，征地拆迁、基础设施建设有序推进，武都吉石坝、宕昌哈达铺现代物流园区建设全面启动。地质找矿取得新进展,完成找矿投入6.5亿元。节能减排完成年度控制目标，重金属污染治理取得新进展。

三、农村经济稳步推进，农民收入持续增加。加大农业种植结构调整和科技推广力度，大力实施粮油优良品种、马铃薯脱毒种薯、全膜双垄沟播、高效农田节水等增产措施，在遭受严重自然灾害的情况下，农业生产依然持续增长，粮食产量达到104万吨，实现六连增。各项惠农政策得到落实，农民享受惠农补贴资金13.5亿元。特色产业提质增效取得新突破，新增核桃、油橄榄、花椒、中药材、茶叶、苹果等特色产业基地面积50万亩，加强以核桃为主的经济林综合管理，完成嫁接换优259万株，防治病虫害66万亩。畜牧养殖向规模化、标准化发展，畜禽出栏和肉蛋奶总量、水产总量均有较大增长。成功举办了武都花椒、宕昌中药材产销对接洽谈会。全市实现特色产业总产值84亿元。扶贫开发步伐进一步加快，完成了12个连片开发试点和145个整村推进项目，减少贫困人口20.29万人。率先在全省建立了乡镇扶贫工作站。输转劳务人员72万人次，实现劳务收入80亿元。实施农村危旧房改造18300户，建成生态文明新农村258个，农村面貌发生了显著变化。双联行动取得阶段性成效，各级联村单位联系贫困村2400多个，近4万名干部职工和社会各界人士联系特困户10万户，为联系村帮办实事5000多件，解决了一些群众长期渴望解决的突出问题。

四、商贸旅游态势良好，消费拉动不断增强。重点景区旅游设施建设进一步加快，创建申报国家4A级景区工作取得新进展。开展系列宣传促销活动，举办了第四届中国乞巧文化旅游节。全市接待游客490万人次，实现旅游综合收入23亿元，旅游对第三产业的带动作用进一步显现。加强了商贸流通工作，武都花椒、茶叶，宕昌中药材，成县核桃“四大专业市场”完成征地拆迁、规划设计等基础性工作， 13个农贸市场建成投用。积极落实鼓励消费政策，深入实施“家电下乡”、“新网工程”，超额完成了 “万村千乡”市场工程建设任务。建立了区域性国家粮食质量监测站，建成农户科学储粮库400套。非公有制经济实现较快发展，私营企业达到3598户，个体工商户达到55688户，实现非公有制经济增加值106亿元，引领了批发零售、餐饮娱乐等第三产业的发展，有力地拉动了社会消费持续活跃。

五、基础设施建设进程加快，发展条件逐步改善。城镇建设力度不断加大，陇南市城市总体规划已经省政府批复，各县县城总体规划编制基本完成，村镇规划编制有序推进。全市完成城镇建设投资43.88亿元。市区长江大道东西延伸项目一期工程路基全线贯通，东江新区开发建设步伐加快，道路、供排水和公益设施建设取得新进展。八县县城实施了一批新区开发和旧城改造重点项目，城市功能不断完善，哈达铺、碧口、盐官等示范性小城镇建设顺利推进，全市城镇化率23.5%。交通建设实现新突破，洛门至礼县、礼县至武都、五马至阳坝公路开工建设，宕昌南河至迭部腊子口公路完成投资1.8亿元，建设农村公路2000公里。水利基础设施投入力度明显加大，实施水利项目331项，总投资8.8亿元。解决了25万人的安全饮水问题，梯田建设和有效灌溉面积持续增加。加强了生态文明建设，推进各类生态工程、城镇绿化和绿色长廊建设，天保工程管护和重点公益林补偿措施得到全面落实。自然灾害监测预警指挥系统建设走在了全省前列，在防灾减灾中发挥了重要作用。电力设施建设得到加强，临江110千伏送变电工程建成投运，农网改造升级项目顺利完成。农村信息化覆盖面进一步扩大，城乡通讯网络服务能力明显增强。

六、财政收支较快增长，融资工作取得实效。积极应对经济运行面临的困难和挑战，抢抓各种有利机遇，全力组织财政收入，切实保障重点支出。全市大口径财政收入首次突破30亿元大关，大口径财政收入、公共财政预算收入同比分别增收6.7亿元和2.95亿元。全市完成公共财政预算支出147亿元，增长33.5%，增支36.9亿元，支出增幅位列全省第二，有力保障了项目建设、民生工程和社会管理等重点支出。国库集中支付等财政改革稳步推进，有效提升了财政科学化管理水平。金融支持力度进一步加大，全市各类金融机构存贷款余额分别达到476.7亿元、243.8亿元，分别增长16%、20%。融资性担保服务体系建设得到加强，新建融资性担保机构11个。大力推进银企合作，签约项目142个，落实贷款67亿元。民生金融取得新成效，市、县区累计注入担保基金1.2亿元，为2.7万名农村妇女发放贷款12亿元，直接或间接带动近30万名妇女创业就业。

七、社会事业协调发展，群众得到更多实惠。全市“两基”成果得到进一步巩固，教育教学改革深入推进，教学质量和办学水平不断提高，高考二本以上录取人数达到3082人，比上年增加360人。实施校舍安全工程79项，新建、改建幼儿园42所。惠及27万名学生的农村义务教育阶段学生营养餐计划全面实施。国家二类城市语言文字工作顺利通过省政府评估验收。卫生事业健康发展，城乡医疗卫生服务体系建设取得新进展，公共卫生服务能力不断增强，新农合参合率稳定在96％以上，年人均筹资水平由230元增加到290元，住院费报销比例由60%提高到75%以上。科技创新和推广工作有新进步，取得科技成果25项，申报专利100件。文化产业发展步伐加快，公共文化服务体系不断完善，文化体制改革完成阶段性任务，地域文化研究和文艺精品创作成果显著，文化产业增加值增速达到33%，市“四馆四中心”完成主体工程，各县区“三馆一中心”建设全面推进，申报华夏文明传承创新区项目120多个，完成了34万户广播电视“户户通”工程，实现了农家书屋行政村全覆盖。全民健身运动广泛开展，成功举办了市第七届运动会。人口和计划生育工作取得新成效，农村符合政策生育率达到87%，人口自然增长率控制在6.8‰以内。全民创业就业工作取得新成效，发放创业就业小额担保贷款15.3亿元，实现以创业带动就业 1.1万人，4000多名大学生通过公开招考实现就业。城乡居民养老保险全覆盖如期实现，城乡最低生活保障标准分别提高13%和16%，五保供养标准提高20%，医疗救助17万人次。开工建设各类保障性住房3803套，住房租赁补贴及时足额发放，住房公积金制度全面推行。安全生产、物价监管和食品药品安全监管措施得到落实。加强和创新社会管理，社会治安防控体系建设有效推进，进一步加强了信访工作，严格落实领导干部信访接待日制度和信访包案责任制，认真抓好信访积案化解工作，上访批次和人数分别下降15%、36%，法制宣传教育和矛盾纠纷排查化解扎实开展，排查化解各类矛盾纠纷7617件，社会大局保持和谐稳定。市委、市政府确定的10件实事全面完成。民族宗教、外事侨务、统计、档案、地方志、气象、“双拥”、人防、民兵预备役和妇女儿童、残疾人、老龄、慈善、红十字会等各项事业取得新进步。

八、改革开放不断深化，发展活力进一步增强。人事制度改革深入推进，出台了引进人才办法，建立了新招考人员到基层工作、市直单位从基层招考工作人员的机制，引进了一批“985”、“211”重点院校人才。医药卫生体制改革取得新进展，全市基层医疗卫生机构和村卫生室全部实施国家基本药物制度和零差率销售。集体林权制度综合配套改革进展顺利，发放林权抵押贷款3亿多元。农村土地流转进一步加快，累计流转土地26万亩。行政管理体制、小城镇及区域协调机制改革、企业改制等深入推进。对外开放和战略合作取得新成果，与中央国家机关青联委员建立了联系对接机制，与安徽淮北、亳州、铜陵等市缔结了友好城市，分别签订了战略合作交流框架协议，与国药集团、三峡集团、山东黄金、海亮集团等一批有实力的企业达成投资协议。成立了陇南浙江商会，与甘肃浙江企业联合会、甘肃安徽商会签订了《经济合作交流和招商引资备忘录》。

各位代表：过去的一年，我们加大向中央有关部委和省直有关厅局的汇报争取力度，使政策运用和项目生成取得了重大进展；我们抓主抓重，推进资源整合，加强战略合作，经济结构进一步优化，资源开发利用水平有较大提升；我们坚持以人为本，关注民生，全面排查落实惠民政策，尽心竭力地兴办民生实事，解决了一些群众普遍关注的突出问题；我们加强政府自身建设，转变工作作风，提高行政效能，坚持用创新的思路、创新的举措推动工作，各级政府的工作水平得到进一步提升。回顾过去，成绩来之不易。这些成绩的取得，是省委、省政府关怀支持的结果，是市委正确领导、科学决策的结果，是市人大、市政协和社会各界监督支持的结果，是全市人民自强不息、艰苦奋斗、顽强拼搏的结果。在这里，我代表市人民政府，向奋战在各条战线上的广大干部群众，向驻陇南人民解放军、武警官兵和中央、省驻陇南单位，向各民主党派、工商联、无党派爱国人士、离退休老干部，向所有关心支持陇南经济发展、社会进步的各界人士表示衷心的感谢和崇高的敬意！

在充分肯定成绩的同时，我们也清醒地认识到，我市发展基础差、经济总量小，贫困面大、贫困程度深的市情还没有根本改变，经济社会发展还面临着许多突出矛盾和问题，主要表现在：保持固定资产投资持续增长的难度大，招商引资的任务艰巨，工作力度还需要进一步加大；农业产业化程度不高，抵御自然灾害的能力不强，农产品流通不畅、质量效益不高，提质增效任务还很艰巨；工业主导产品特别是铅锌等有色冶金产品价格持续低迷，销路不畅，工业企业效益不高，进一步做大做强的压力比较大；发展环境虽然有了一定改善，但征地难、拆迁难、建设项目落地难的问题仍然比较突出；社会管理基层基础工作比较薄弱，各种利益需要和诉求问题不断增多，做好新时期群众工作难度较大，维护社会稳定任务非常繁重；对照中央转变工作作风、密切联系群众的要求，进一步转变政府职能、改进工作作风、提高工作水平的任务仍然较重。对这些问题，我们将在今后的工作中采取有效措施，认真加以解决。

2013年主要工作任务

各位代表：今年是深入实施“十二五”规划和秦巴扶贫规划的关键一年，做好今年的工作具有十分重要的意义。今年政府工作的总体要求是：深入贯彻党的十八大精神和省委、省政府重大决策部署，紧紧围绕市委总体工作思路，以项目建设和扶贫开发为总揽，着力抓好交通建设、城镇建设、生态建设，着力做大工业经济总量、提升农业产业化水平、加快发展文化旅游产业，着力改善民生、优化发展环境、维护社会稳定，推动经济社会转型跨越发展。

经济社会发展的主要预期目标是：生产总值增长14%；固定资产投资增长37%；规模以上工业增加值增长21%；财政收入增长20%；社会消费品零售总额增长18%；城镇居民人均可支配收入增长16%；农民人均纯收入增长17%；城镇登记失业率控制在4%以内；人口自然增长率控制在6.8‰以内；居民消费价格涨幅控制在3.5%；全面完成年度节能减排目标任务。

各位代表：今年已过去了4个多月时间。4个多月来，我们按照市委的总体工作部署，突出重点，狠抓落实，克服干旱和低温霜冻灾害的不利影响，各项工作已经取得了初步的成效，经济社会发展实现了良好开局。一季度全市生产总值完成56.4亿元，同比增长11.8%；完成固定资产投资36.99亿元，增长30.1%；完成大口径财政收入10亿元、公共财政预算收入5.8亿元，分别增长18.6%、60.8%；实现社会消费品零售总额14.3亿元，增长15.3%；农民人均现金收入948元，居民人均现金可支配收入3856元，分别增长20.6%、10.8%。

为了确保完成全年各项目标任务，后几个月我们将着力抓好以下工作：

一、全力抓好项目建设，保持投资稳定增长

围绕贯彻“3341”项目工程，抓好政策运用和项目争取工作，实施一批带动力强的大项目、好项目。一是加快重大项目建设。做好协调服务工作，妥善解决重大项目建设拆迁占和施工保障等问题，确保武罐高速公路年内实现全线通车，加快兰渝铁路建设，完成计划投资30亿元，成武高速公路、十天高速公路分别要完成计划投资34亿元、48亿元，抓好成州民用机场征地拆迁、“三通一平”和试验段建设，完成年度建设任务，力争渭源至武都高速公路年内开工建设，完成武都至九寨沟高速公路预可研和十天高速两当连接线可研报批工作，积极争取早日启动天平铁路南延线、阳平关至武都至九寨沟铁路等项目前期工作。实施市列重点项目200个，抓好礼县秦文化产业园、武汉凯迪徽县生物质能源林基地建设、西和果蔬中药材批发市场等重点项目。二是加大项目资金争取力度。紧盯国家宏观调控政策，找准争取项目的结合点，全力做好汇报争取工作。特别要依托省政府《关于支持陇南市加快经济社会发展的意见》、“3341” 项目工程和秦巴扶贫开发、“两江一水”综合治理、生态示范市建设、华夏文明传承创新区建设等政策规划，进一步加大争取项目资金的力度，努力把各类规划和政策机遇变成项目和资金，为进一步加快发展提供有力支撑。三是进一步加强项目论证储备工作。邀请委托科研院所，聘请有关专家学者，紧紧围绕“3341”项目工程，在园区建设、循环经济、交通基础设施、生态建设、环境保护、工业支柱产业、农林特色产业、文化旅游产业等方面，深度谋划一批稳增长、促发展的项目，充实完善项目库，储备千万元以上项目800个以上，亿元以上项目600个以上，为争取项目和招商引资打好基础。四是坚持不懈地抓好招商引资。全面落实招商引资工作责任制和各项优惠政策，进一步优化招商引资环境，把走出去、请进来、定点招商、小分队招商作为招商引资的主要方式，加大精准招商引资力度，加强与友好城市、重点企业、商会组织、陇南籍在外企业家的联系对接，鼓励市内企业与国内500强企业、民营500强企业开展各种形式的战略合作，引进更多的合作企业和项目，努力增加非国有投资。力争签约资金250亿元以上，到位资金130亿元以上。

二、着力加快基础设施建设，进一步消除瓶颈制约

抢抓省上实施三大基础建设的机遇，全力加快交通设施、城乡基础设施和水利、通讯、能源设施建设。一是加强交通基础设施建设。争取开工建设一批国省干线公路城镇过境段改造工程，加快县乡公路改造升级步伐，加强出口路、断头路、联网路建设，建成宕昌至迭部、洛门至礼县、两当至天水等重点公路，加快建设礼县至武都、徽县县城至火车站公路，抓好通乡、通村公路建设，实施1000公里建制村通畅工程，实现94%的乡镇和40%的建制村通沥青路或水泥路，基本实现建制村村村通公路。支持发展公共交通，加快城乡公交一体化进程。二是加快城镇建设步伐。面对区位优势不断提升的有利机遇，高点定位和谋划城镇建设，充分发挥城镇化对扩大内需的带动作用。不断完善城镇建设规划体系，加快市区东江新区、钟楼滩新区、吉石坝新区开发建设，年内建成长江大道东西延伸一期工程，加大火车站项目征地拆迁力度，统筹解决资金难题，加快行政中心建设进度，抓好市政广场、路网工程和安置区建设，扩大城市框架和发展空间。运用市场机制，抓好八县县城道路、供排水、垃圾污水处理等136个市政项目，不断完善城镇功能。深入推进以21个示范镇和公路沿线建制镇为重点的小城镇建设，促进县城、建制镇协调发展，使城镇化率达到26%。加强城镇管理，提高环境卫生、市容市貌、交通秩序管理水平，着力解决卫生脏乱差、违章建设等突出问题，倡导文明生活理念，提高居民素质，提升城市品位，创造健康、文明、和谐的人居环境。三是加快水利基础设施建设。积极争取“两江一水”综合治理规划尽快获得国家批复并启动实施，加快推进重点河流治理，实施一批堤防工程，加快病险水库除险加固，加强城镇防洪体系建设；抓好山丘区特色农业节水灌溉示范区项目，力争把8县1区全部纳入全国农田水利重点县项目实施范围，新增高效节水灌溉面积10万亩。四是全面推进能源、通讯建设。加强电源电网建设，加快建设武都洛塘110千伏送变电工程，争取开工建设两当、哈达铺、武都阶州等110千伏送变电工程，继续抓好农网改造工程，推进水电新农村电气化县建设，加快实施小水电代燃料生态保护工程、农村水电增效扩容改造工程，积极开发利用生物质能、太阳能、风能等清洁能源。进一步加快通讯建设，提高保障服务水平。

三、大力推进扶贫开发，做大做强富民特色产业

坚持以扶贫开发统揽农村工作全局，认真实施秦巴扶贫规划，加快发展核桃、花椒、油橄榄、中药材等农业特色产业，努力推动农村经济快速发展。一是加快改善生产生活条件。积极推进秦巴片区扶贫规划实施，突出扶贫重点，瞄准扶贫对象，着力抓好高寒阴湿区、深山林缘区、半山干旱区和少数民族聚居区等特困片区扶贫攻坚，坚持山水田林路综合治理，加大梯田建设力度，进一步实施好退耕还林工程，不断改善生产条件，增强自我发展能力。按照整片整流域开发模式，实施150个整村推进项目、9个整乡推进项目。加快农村改厕、改圈、改灶，建设农村户用沼气7000户。二是全面提升农业产业化经营水平。要以扶贫项目资金为支撑，以市场为导向，充分发挥特色农业资源比较优势，扶持引导农民加快核桃、花椒、油橄榄、中药材等为主的特色产业标准化基地建设，实施品种改良、配方施肥、病虫害防治等实用技术推广应用，促进特色产业提质增效。大力推进白水江、白龙江沿岸油橄榄基地建设，争取实施50万亩油橄榄水利配套项目，为把武都打造成全国橄榄之城打下良好基础。加大中药材产业引进战略合作的力度，扩大GAP种植面积。加快发展马铃薯、蔬菜、苹果、茶叶和畜禽产业，启动12个市级现代特色农业示范区建设，力争白龙江沿岸10万亩蔬菜基地立项实施，争取武都现代农业示范区升格为国家级园区。大力扶持独一味新药研究、徽县银杏生产线、宕昌当归油萃取等项目，培育发展龙头企业，提高中药材产业开发水平。抓好100个市级农民专业合作社示范社建设，力争全面建成核桃、花椒、茶叶、中药材四大专业市场并启动运营，有效解决农产品卖难问题。培育认定5户以上省级龙头企业，培育油橄榄、核桃、花椒等特色农产品知名品牌，提高群众收入。加快土地流转，促进土地集约经营和特色产业规模化发展，实现土地流转面积翻一番。三是坚定不移地抓好农业生产。稳步推进农业种植结构调整，加强农业综合管理和农业技术服务体系建设，全面落实粮油高产创建措施，积极推广全膜双垄沟播等适用技术，加快马铃薯种薯扩繁基地建设，大力发展优质高效农业，提高农产品产量和质量，力争粮食总产达到104万吨以上。四是大力发展劳务经济。把劳务输转作为农民增收的重要措施，加强劳务基地建设，充分发挥各级劳务机构、中介组织和劳务经纪人、输转带头人的作用，努力扩大有组织输转规模。整合各类教育培训鉴定资源，大力开展外出务工人员技能培训和技能鉴定，使符合条件的务工人员都能取得职业资格证书。提高劳务输转信息化服务水平，实现农民工跟踪服务，做好农民工维权保障工作。大张旗鼓地表彰奖励劳务带头人、经纪人和中介组织，在全社会倡导崇尚创业、致富光荣的理念。建立市场化运作的农业专业服务组织，为外出务工家庭提供良好服务。完成劳务输转73万人次，创收94亿元以上。力争全市贫困人口减少25万人。深入开展“联村联户、为民富民”行动，落实帮扶项目，兴办实事好事，培育富民产业，着力破解农村发展难题。完善新农村建设规划，坚持向上争取项目资金与调动农民群众积极性相结合，形成多元投入机制，高标准、高质量地推进新农村建设，建成210个生态文明新农村。

四、强力加快工业化进程，不断壮大经济总量

着力打造以徽成经济开发区和循环经济示范区为重点的经济战略平台，改造提升传统产业，积极发展有色冶金、农特产品加工、现代制药、建筑建材、水电能源等产业，不断做大工业总量。一是加快工业项目建设。实施市列重点工业项目32个，建成投产紫金矿业尾渣综合利用、红川酒业扩能技改等重点项目，加快厂坝公司300万吨扩能技改、金徽矿业郭家沟铅锌矿采选、成县祁连水泥二期扩建等项目建设进度，抓紧做好厂坝公司20万吨锌冶炼、青岛红星30万吨钡盐、文县阳山金矿、凯迪生物质发电等项目前期工作，力争早日开工建设。积极发展循环经济，实施一批节水节能、资源综合利用项目，重点抓好成县祁连水泥余热发电、金徽酒业废弃物综合利用等项目。二是扶持重点企业发展壮大。认真落实扶持优惠政策，全力支持15户重点企业的生产经营，确保重点企业工业增加值占全部规模以上工业增加值的比重达到65%以上。加快新建投产企业和规下企业入规步伐，争取西和大桥金矿、后川坝金矿、两当金润玉石等14户企业进入统计名录。依托优势水电资源，用好高载能产业电价补贴政策，扶持发展高载能产业，力争硅电一体化开发取得较大突破。加强资本运作，加快金徽酒业上市步伐。落实优惠政策，加大对中小企业扶持力度。三是不断加大矿产资源勘探和资源整合力度。开展大规模的地质勘查专项整治活动，完成9个重点区域的非金属矿产资源调查工作。积极构建地质找矿新机制，引入大型企业集团与现有探矿企业开展战略合作，走勘探、采选、冶炼、深加工一体化的道路，提高矿产资源开发利用水平。把资源整合作为推进工业发展的重要抓手，已有的资源配置按照扶大扶强的原则，优先向大企业倾斜，发展产业集群，做大做强优势产业。四是推进工业园区建设取得新突破。按照“一区多园”运行模式，进一步加快徽县工业集中区整体纳入西成经济开发区扩区步伐。加强徽县工业集中区以道路、厂房为主的基础设施建设，抓好资金筹集、土地报批和征地工作，加快入园项目建设进度，加强园区招商引资，引进工业项目10个以上。加强武都吉石坝、宕昌哈达铺等现代物流园区基础设施建设，力争取得新进展。

五、提升旅游商贸整体水平，带动第三产业快速发展

加快旅游规划编制工作，完成3个县的旅游总体规划、5个景区详细规划等规划编制工作。创新旅游发展理念，引入大型旅游集团、民间资本、社会资本，采取各种方式参与旅游资源开发，加快武都万象洞、文县天池、徽县三滩、宕昌官鹅沟等旅游景区重点项目建设，抓好康县阳坝、宕昌哈达铺两个旅游示范镇和16个旅游示范村建设。年内力争创建4A级旅游景区2-3处。深入挖掘整理革命遗存，全面建成两当起义纪念馆及旧址改造等项目，挖掘宕昌国、仇池国等历史文化资源以及乞巧、白马民俗等非物质文化遗产，加大哈南寨等历史文化名村保护开发力度，发展名镇、古寨特色旅游，推动文化与旅游深度融合。创新旅游宣传促销方式，开展“美丽乡村”等系列宣传活动，加大景区景点宣传力度，打造精品线路和旅游品牌，不断提高陇南旅游知名度和影响力，全年接待游客615万人次，旅游综合收入达到29亿元。进一步加强商贸流通工作，加快流通体系和商贸设施建设，培育、引进大型连锁商贸企业，为各类商品和生产要素“大进大出”创造条件。推进农超对接等产销衔接模式，实施好 “万村千乡”市场工程。加快发展仓储、配送、物流、保险、广告、咨询、信用担保、文化创意等现代服务业，促进第三产业稳定增长。

六、全面推进生态建设，努力构建生态安全屏障

科学编制生态建设规划，明确划分自然恢复区、封山禁牧区、综合治理区和人工造林区，把生态文明建设与“两江一水”区域综合治理、生态示范市工程结合起来，多措并举，统筹推进整片整流域建设。一是扎实推进林业生态建设。把生态效益和民生效益作为绿化造林的根本出发点，把造林绿化与特色产业、风貌改造、文明长廊和新农村建设相结合，坚持因地制宜，突出地方特色，创新造林模式，提高造林成效，为建设美丽陇南打下坚实基础。完成造林30万亩。深入实施小流域综合治理、水土保持、封山禁牧等工程，巩固退耕还林成果，争取国家启动长江防护林三期工程。二是大力开展环境污染治理。加强重点区域污染防治、排污企业综合治理和重金属污染治理，抓好西和县重金属污染治理示范县建设，完成重点环境治理项目，推进58个村的农村环境连片整治工作，争取国家将陇南列为全国尾矿库治理示范市。三是进一步加强地质灾害综合治理和防灾减灾工作。争取国家启动易灾地区生态环境综合治理规划，加快实施白龙江、北峪河、西汉水等重点区域滑坡、泥石流综合治理项目。加大资源整合力度，改造提升自然灾害预警指挥系统，更好地发挥预警预报和应急指挥作用。加强农业气象服务体系和农村气象灾害防御体系建设，加快实施新一代天气雷达监测系统建设项目，填补我市雷达监测方面的空白，切实提高防灾减灾水平。

七、做好财税金融工作，增强服务发展能力

充分发挥财政、信贷调节作用，加大对经济社会发展的支持力度。一是努力保持财政收入稳定增长。加强财源建设，继续落实各项产业发展优惠政策，巩固支柱财源，扩大新兴财源，培育后续财源，建立多元化的财源体系。优化财政收入结构，强化重点行业企业和重大建设项目等重点税源监管，加强零散税收、非税收入征管，加大税收稽查力度，做到公平税赋、应收尽收。二是提高公共财政保障水平。调整优化财政支出结构，严格控制一般性支出，把保障重点放在“三农”、 社会事业、民生实事、主导产业和重点项目上，集中财力办大事。稳步推进部门预算等重点领域财政管理体制改革，发挥财政资金使用效益。认真落实财政结构性减税政策，积极支持中小微企业发展。三是努力破解融资难题。发挥国有资本对配置市场资源的引导作用，完成市城乡发展投资公司组建工作，加快推进市政府投融资平台建设。支持各类金融组织设立分支机构和服务网点，鼓励发展村镇银行、小额贷款公司、资金互助社等新型农村金融组织，激发融资活力。鼓励银行业金融机构开展经营机制、金融产品和服务创新，建立和完善中小微企业金融服务体系。完善银地银企合作机制，通过设立“市长金融奖励基金”、财政贴息等措施，提高金融机构支持“三农”和小微企业发展的积极性。进一步拓展担保范围，扩大林权抵押贷款放贷规模，解决“三农”和小微企业担保难的问题。实施好 “双联惠农”贷款项目，力争贷款额度达到15亿元以上。大力培育和引进信用担保机构，完善资本金补充和风险补偿机制。通过财政奖励等方式积极支持符合条件的企业通过上市和发行债券进行直接融资。

八、统筹发展文化等社会事业，不断拓展民生保障领域

在华夏文明传承创新区建设总体布局中，建设特色文化大市，开展文化资源普查，落实“十三板块”工作任务，深入推进文化体制改革，规划实施一批文化产业项目，积极培育文化产业，加快先秦文化、西狭文化等地域文化的挖掘整理，论证建设具有地域特色的文化产业园区，促进文化企业向园区聚集。采用多种方式，加大对陇南红色文化、民俗文化、地域文化、历史文化等文化资源的宣传力度，增强陇南文化的影响力和传播力。建成“四馆四中心”并投入使用。大力发展教育事业，抓好义务教育学校标准化建设和中小学布局调整，推进县域义务教育均衡发展。加强教师队伍建设，抓好中小学校舍安全工程和农村薄弱学校改造，改善办学条件，落实各类教育惠民政策。提升高中教育质量，陇南一中积极创建省级示范性高中。扩大职业教育办学规模，支持礼县职业中专创建国家级重点中等职业学校，加快发展学前教育、特殊教育，积极创建省级示范性幼儿园，支持陇南师专转型发展。大力推进技术创新，学习推广科技成果，深入推进科技特派员创业行动，充分发挥科技人才的作用。加快发展卫生事业，进一步加强城乡医疗服务体系和卫生队伍建设，加强疾病防控和妇幼保健工作，大力发展中医药事业，积极推进县级公立医院综合改革，提高公共卫生服务水平。巩固和完善新型农村合作医疗制度，提高新农合财政补助标准和保障水平。深入开展计生工作“精细管理规范提升年”活动，健全市县乡村四级人口计生服务网络，加强流动人口服务管理和人口计生信息化建设，落实各项优惠政策，努力稳定低生育水平。加快城乡体育设施建设，广泛开展全民健身活动，提高竞技体育水平，积极备战2014年全省第十三届运动会。大力推进全民创业，落实各种扶持政策，鼓励大中专毕业生、退伍军人和妇女创业就业，抓好省、市组织的基层事业单位招聘考试工作，多渠道促进普通高校毕业生就业，确保新增城镇就业2.86万人以上。加快解决城乡低收入群体住房困难，建设保障性住房926套，争取实施农村危旧房改造3万户。完善社会保障体系，加快被征地农民纳入社会养老保险步伐，建立健全大病补助医疗保障制度，推进基本医疗保险制度城乡一体化改革，建立城乡低保动态管理机制，提高城乡低保补助标准，重视和支持慈善事业发展，进一步提升社会保障和救助水平。全面贯彻落实党的民族宗教政策，促进民族宗教事业发展。加强民兵预备役建设，支持驻陇南部队建设，坚持开展全民国防教育，深化双拥共建工作。重视发展老龄、残疾人、档案和红十字会事业，支持工会、妇联、共青团等群众团体的工作，维护好职工、妇女儿童和青少年的合法权益。

持之以恒地改善民生办实事，2013年重点办好10件实事：⑴解决25万农村人口饮水安全问题。⑵实施易地扶贫搬迁1.2万人。⑶建设100个未通公路行政村的道路通达工程。⑷加快建设武都五马至康县阳坝扶贫公路，年内完成路基工程。⑸建设或升级改造12个县乡农贸市场。⑹建设50所乡镇幼儿园。⑺建设100所标准化村卫生室。⑻在武都旧城区建设3座人行天桥。⑼完成1000个城市公共视频监控点建设任务。提高市、县广播电视覆盖率。⑽对城乡已婚妇女进行“两癌”检查。

九、全面加强社会管理，促进社会和谐稳定

进一步加强社会管理信息化建设，加快建立自然灾害、宣传文化、社会管理三大信息综合服务系统。加强政府应急指挥平台建设，完善应急处置机制，提高应急管理水平。抓好群众来信来访办理工作，坚持领导干部接待群众来访、包案处理信访问题等制度，畅通群众诉求表达渠道，切实解决群众合理诉求和切身利益问题，着力化解征地拆迁、涉法涉诉等信访积案，从源头上预防集体上访和越级上访。加快推进法治陇南建设，深入开展法制宣传，坚持公正廉洁执法，维护社会公平正义。建立健全维护群众权益机制，开展社会稳定风险评估，强化矛盾纠纷排查调处工作，努力化解社会矛盾。加强社会管理综合治理，创新社会管理，深化平安建设，构筑立体化的社会治安防控体系，依法严厉打击违法犯罪，有效解决影响公众安全感的突出问题。加强社区建设，提升社区自治水平，发挥好社区管理服务、社会保障、文化娱乐、维护稳定等方面的作用。严格落实安全生产责任，加强安全生产基层基础建设，抓好矿山、交通运输、建筑工程等事故多发领域的专项整治，严防重特大事故发生。整顿和规范市场秩序，全力保障生产生活必需品的储备供应和质量安全,加强物价监测调控，保持物价基本稳定。

各位代表：推动跨越发展，实现富民强市，人民寄予厚望，深感责任重大。我们必须以敢于担当的勇气，进一步加强政府自身建设，认真贯彻中央、省委和市委关于改进工作作风、密切联系群众的各项规定，勤勉工作，不辱使命，努力建设为民、清廉、务实、高效的服务型政府。一是加强理论武装，建设学习政府。牢固树立终身学习理念，刻苦学习履行职能必需的知识，更新完善知识结构，强化战略思维、创新思维、辩证思维，不断提高科学决策能力和解决实际问题的能力。要紧跟时代步伐，不断研究发展中的新情况、新形势，积极破解前进中的新问题、新矛盾，着力增强工作的主动性、预见性和创造性，努力提升用好政策、抢抓机遇和推进跨越发展的水平，真正把学习创新的成效体现在攻坚克难上，落实到具体工作中，贯彻于发展实践中。二是加快职能转变，建设责任政府。认真履行政府职能，理顺政府与市场、与社会之间的关系，把市场能办的事放给市场，管好政府应该管的事。集中精力谋大事，集中时间抓要事，集中力量解难事，着力解决改革发展中的重点问题，妥善处理事关人民群众切身利益的具体问题。坚持正确的政绩观，多做打基础、利长远的工作，多办顺民心、惠民生的实事，使人民群众在发展中得到更多实惠。加大行政问责力度，严格执行首问责任制、限时办结制和责任追究制，扩大政务公开范围，加快电子政务建设，提高政务服务质量和水平。三是坚持依法行政，建设法治政府。严格依照法定权限履行职责，管理经济社会事务，规范行政行为，把依法行政贯穿到政府工作的各个领域、各个环节。健全行政决策机制，完善重大事项集体决策、专家咨询、社会公示与听证制度，提高依法、科学、民主决策水平。自觉接受人大及其常委会的法律监督和政协民主监督，认真办理人大代表建议和政协委员提案，广泛听取人大代表和政协委员的意见建议，为人大代表和政协委员参政议政、发挥才智创造良好条件。四是狠抓工作落实，建设效能政府。把提高执行力和抓落实的能力作为各级政府作风建设的落脚点，进一步健全完善抓落实的相关制度，加强重点工作督查，不断完善和坚持责任目标考核和奖罚制度，严格实行一票否决，用制度保障工作落实。大力改进文风会风，少发文开短会，勤办事办实事，把时间和精力更多地用到发展经济、改善民生上来。政府部门要强化服务意识，下大力气转变工作作风，树立定了就办、办就办快、办就办好的良好风气，提升工作效能和服务质量。五是坚持清正清廉，建设廉洁政府。认真落实廉政建设责任制，加强政府机关工作人员廉洁从政教育，健全风险预警和权力运行监控机制，规范土地开发、政府采购、项目资金安排、国有资产处置和重大工程建设招投标管理，进一步健全行政监察、审计监督等内部监督机制，发挥好监察、审计部门的职能作用，严肃查处违法违纪案件，坚决纠正损害群众利益的不正之风。牢固树立过紧日子的思想，实行财务公开，坚持厉行节约，坚决杜绝讲排场、摆阔气等铺张浪费行为。严格落实廉洁自律各项规定，加强对亲属和身边工作人员的教育和约束，做到干部清正、政府清廉、政治清明。

各位代表：陇南的发展已经站在新的历史起点上，梦想孕育奇迹，万事成于艰辛。功崇惟志，业广惟勤。让我们在省委、省政府和市委的坚强领导下，在市人大、市政协的监督支持下，紧紧依靠和团结全市各族人民，同舟共济，开拓进取，埋头苦干，奋勇争先，为建设幸福美好新陇南而努力奋斗！
